# STAT3 in Cancer—Friend or Foe?

**DOI:** 10.3390/cancers6031408

**Published:** 2014-07-03

**Authors:** Hai-Feng Zhang, Raymond Lai

**Affiliations:** 1 Department of Laboratory Medicine and Pathology, University of Alberta, Edmonton, AB T6G 1Z2, Canada; E-Mail: haifeng4@ualberta.ca; 2 The Key Laboratory of Molecular Biology for High Cancer Incidence Coastal Chaoshan Area, Shantou University Medical College, Shantou 515041, Guangdong, China

**Keywords:** STAT3, oncoprotein, tumor suppressor, STAT3β, prognostic marker, therapeutic target

## Abstract

The roles and significance of STAT3 in cancer biology have been extensively studied for more than a decade. Mounting evidence has shown that constitutive activation of STAT3 is a frequent biochemical aberrancy in cancer cells, and this abnormality directly contributes to tumorigenesis and shapes many malignant phenotypes in cancer cells. Nevertheless, results from more recent experimental and clinicopathologic studies have suggested that STAT3 also can exert tumor suppressor effects under specific conditions. Importantly, some of these studies have demonstrated that STAT3 can function either as an oncoprotein or a tumor suppressor in the same cell type, depending on the specific genetic background or presence/absence of specific coexisting biochemical defects. Thus, in the context of cancer biology, STAT3 can be a friend or foe. In the first half of this review, we will highlight the “evil” features of STAT3 by summarizing its oncogenic functions and mechanisms. The differences between the canonical and non-canonical pathway will be highlighted. In the second half, we will summarize the evidence supporting that STAT3 can function as a tumor suppressor. To explain how STAT3 may mediate its tumor suppressor effects, we will discuss several possible mechanisms, one of which is linked to the role of STAT3β, one of the two STAT3 splicing isoforms. Taken together, it is clear that the roles of STAT3 in cancer are multi-faceted and far more complicated than one appreciated previously. The new knowledge has provided us with new approaches and strategies when we evaluate STAT3 as a prognostic biomarker or therapeutic target.

## 1. The Normal Functions of STAT3

STAT3 belongs to a family of transcription factors that transduces the cellular signals from a host of cytokines and soluble growth factors such as the IL-6 family cytokines, epidermal growth factor and platelet-derived growth factor [1,2]. In the canonical pathway, ligation of cytokines to their respective cell-surface receptors induces dimerization and auto-phosphorylation of various tyrosine residues of Janus kinases (JAKs), which then serve as the docking sites for the inactive, monomeric STAT3 molecules. The JAK-bound STAT3 molecules are then phosphorylated by JAKs at the tyrosine residue 705 (STAT3^Y705^), a crucial event for the subsequent dimerization and activation of STAT3. Subsequently, the phosphorylated STAT3 (pSTAT3) molecules form homodimers, which migrate to the nuclei where they bind to the promoters of various target genes and regulate their transcriptions [1,2]. Many STAT3 targets, such as Survivin, Cyclins and the Bcl-2 family proteins, are known to promote cell proliferation and survival [1,3,4]. In recent years, accumulating evidence has suggested the existence of the non-canonical pathway, in which the functions of STAT3 are independent of the phosphorylation of STAT3^Y705^ or its nuclear translocation [5]. 

STAT3 has important and diverse biological functions in normal cells, and details can be found in a number of excellent reviews [6,7,8]. Briefly, its biological importance is highlighted by the observation that *STAT3* gene ablation in mice results in embryonic lethality that occurs 6–7 days after inception [9]. Studies using conditional *STAT3* knockout mice have provided evidence that STAT3 is required for the development and differentiation of various tissue types, such as the skin, immune system, liver, mammary gland, thymus and nervous system [6]. For example, ablation of *STAT3* in keratinocytes was found to impair migration of keratinocytes and skin remodelling [10]. In another study, deletion of *STAT3* in the mammary glands was found to suppress apoptosis of the glandular epithelial cells and lead to delayed glandular involution [11]. STAT3 is critical to the development and biology of T-cells. In one study in which STAT3 was conditionally ablated in all stratified epithelia including the thymic epithelia, there was a dramatic increase in apoptosis in thymocytes; in addition, STAT3-depleted thymocytes were more susceptible to apoptosis induced by dexamethasone and γ-irradiation [12]. In another study, STAT3 was shown to be important in mediating the anti-apoptotic effect of IL-6 in the presence of a low-serum culture environment [13].

## 2. The Oncogenic Potential of STAT3

### 2.1. An Overview

The oncogenic roles of STAT3 have been extensively published and reviewed in the literature [1,2,3,4,5], and only a brief summary will be provided here. Some of the first evidence supporting the oncogenic role of STAT3 came from studies using *STAT3C*, which is a constitutive active STAT3 mutant construct [14,15,16]. STAT3C contains two cysteine substitutions at the residues A661 and N663, leading to the formation of disulfide bridges between two STAT3 molecules and mimicking STAT3 homodimerization that occurs in the normal activation process [14]. It has been demonstrated that STAT3C can effectively induce malignant transformation [14,15,16], and that STATC can transcriptionally increase the expression of many genes that are important in promoting cellular proliferation, resistance to apoptosis, angiogenesis, immune evasion, invasion and metastasis, all of which are hallmarks of cancer [1,2,3,4,5]. Inappropriate activation of STAT3 has been revealed in various types of solid and hematological cancers, and blocking the STAT3 signaling pathway by various means is effective in killing cancer cells in many experimental models [3,4,17]. More recently, STAT3 has been implicated in the self-renewal of cancer stem cells [18,19,20,21,22]. For instance, it was found that STAT3C cooperates with the embryonic stem cell marker Sox2 to initiate the malignant transformation process in esophageal basal cells [22].

To reinforce the concept that STAT3 is oncogenic when it is inappropriately or constitutively activated, many laboratories have shown that the dominant negative STAT3 mutant construct, often labeled STAT3-DN in the literature, can effectively mediate cell cycle arrest and/or induce apoptosis in cancer cells [23,24,25]. STAT3-DN is generated by substituting the Y705 residue with phenylalanine, and thus, STAT3 cannot be phosphorylated. STAT3-DN is believed to exert its biological effects by competing with the endogenous STAT3 molecules for the binding sites on JAKs and other STAT3 activating proteins, thereby limiting the activation of endogenous STAT3. Many studies that had used other means of STAT3 inhibition (e.g., siRNA and small peptides) produced similar results, as reviewed by Wang *et al.* [26].

Constitutive activation of STAT3, which has been demonstrated in a broad spectrum of solid and hematological cancers, often correlates with an unfavorable prognosis in cancer patients [2,4,27]. A few recent publications are used to illustrate this point. In a cohort of 262 gastric tumor samples, Xiong *et al.* found that patients carrying tumors with phosphorylated STAT3^Y705^ (or pSTAT3) expression had significantly shorter overall survival compared with those carrying tumors without pSTAT3 [28]. In a study of colorectal cancer, pSTAT3 expression was found to significantly correlate with the depth of tumor invasion, status of lymph node, metastasis and tumor stage [29]. Huang *et al.* also reported that a high expression of *STAT3* mRNA or pSTAT3 protein significantly correlates with a short overall survival and event-free survival in a cohort of patients with diffuse large B-cell lymphoma [30]. STAT3 activation was found to predict a worse clinical outcome in many other types of cancer, such as cervical cancer [31], esophageal squamous cell carcinoma [32,33], head and neck squamous cell carcinoma [34,35] and thymic epithelial cancer [36].

### 2.2. Mechanisms Underlying the Constitutive Activation of STAT3 in Cancer

In normal cells, physiologic activation of STAT3 in response to extracellular signals such as growth factors and cytokines is a transient event, largely due to the existence and operation of various negative feedback mechanisms [37,38]. The constitutive activation of STAT3 in cancer cells represents a biochemical aberrancy, and there are at least four mechanisms shown to contribute to this abnormality: (1) loss of the negative regulation of STAT3; (2) excessive stimulation of STAT3; (3) positive feedback loops that sustain persistent STAT3 activation; (4) somatic mutations that confer a hyperactive property to STAT3.

#### 2.2.1. Loss of the Negative Regulation of STAT3

To avoid inappropriately sustained activation of STAT3, there are multiple negative regulators that can promptly silence STAT3 signalling. The suppressors of cytokine signaling (SOCS) and protein tyrosine phosphatases (PTPs) are two families of proteins that carry out this important function [38,39,40]. The SOCS family of proteins is made up of eight members in mammalian cells, including SOCS1-7 and the cytokine-inducible SH2 protein, all of which have been shown to regulate cell growth, differentiation and survival, and to modulate dendritic cell functions, inflammatory response and hematopoiesis [41]. These proteins interact with the kinase domain of JAKs, and inhibit signal transduction by competing with various signaling molecules (such as STATs) for the docking sites on the receptors. Via their SOCS box domain, SOCS proteins also interact with E3 ubiquitin ligases and thereby promote the ubiquitin-dependent degradation of their targets [41]. Normally, the expression of the SOCS proteins is under tight control, with robust induction by a wide spectrum of cytokines and growth factors to prevent the extracellular signals from over-firing and to ensure that the physiological responses are not excessive [41]. Thus, upon cytokine stimulation, the activated JAK/STAT3 signaling pathway promotes the expression of SOCS3, which serves as an important negative feedback mechanism to prevent over-activation of this pathway. 

The homeostasis of STAT3 phosphorylation and activation is frequently disrupted in cancer cells due to the loss of SOCS expression [42]. In this regard, epigenetic silencing of *SOCS3* has been found in various types of cancer, including lung cancer, head and neck squamous cell carcinoma, hepatocellular carcinoma and Barrett-associated esophageal adenocarcinoma [42]. Experimental results have revealed that loss of SOCS3 indeed contributes to the activation of STAT3 in cancer cells, thereby promoting their proliferation, survival and motility [43,44,45,46]. SOCS1, another member of the SOCS family, is also frequently silenced by gene methylation, and this biochemical aberrancy has been shown to contribute to constitutive STAT3 activation in a wide range of cancer types [44,47,48,49,50].

PTPs belong to a large family of proteins with >100 members, which is responsible for counteracting the effects of protein tyrosine kinases and maintaining the overall homeostasis of protein tyrosine phosphorylation. PTPs are known to dephosphorylate and thus inactivate the JAK/STAT3 signaling [51]. Similar to the SOCS proteins, many PTPs involved in the regulation of the JAK/STAT3 signaling are repressed or silenced in cancer cells. For example, SHP-1, a member of the tyrosine phosphatases highly expressed in normal lymphoid cells, is lost in many types of hematologic malignancies due to epigenetic silencing [49,52,53,54]. Loss of SHP-1 has been shown to directly contribute to the constitutive activation of STAT3 in these cancer types, including ALK-positive anaplastic large cell lymphoma, chronic myeloid leukemia and multiple myeloma, since gene transfection of *SHP1* in these cells can substantially decrease the level of STAT3 activation [49,52,53,54]. Interestingly, loss of SHP-1 in ALK-positive anaplastic large cell lymphoma is a direct consequence of the constitutive activation of STAT3 in these cells, as STAT3 plays a key role in promoting gene methylation and silencing of *SHP1* [53]. Thus, loss of SHP-1 and the constitutive activation of STAT3 form a vicious cycle in these lymphoma cells.

Other than the SOCS members and PTPs, PIAS3 (*i.e.*, protein inhibitors of activated STAT3) is also known to inhibit STAT3 by reducing its DNA-binding and ability to regulate gene transcription. The expression of PIAS3 has been shown to be reduced in glioblastoma, and this finding correlates with an elevated level of STAT3 activation and increased cell proliferation [55]. Transfection of *PIAS3* into lung cancer cell lines can suppress cell proliferation and enhance the sensitivity of cells to chemotherapeutic drugs [56]. In parallel with this concept, Kluge *et al.* found an inverse correlation between the expression levels of PIAS3 and pSTAT3 in lung squamous cell carcinomas [57]. 

#### 2.2.2. Excessive Stimulation of STAT3

Cancer cells and some of their surrounding inflammatory cells have been shown to produce and release various soluble factors (notably cytokines) into the tumor microenvironment, such that STAT3 in the cancer cell population is activated excessively and continuously. Cytokines involved in these autocrine or paracrine stimulatory pathways include IL-6, IL-10, IL-11, IL-21, IL-23, leukemia inhibitory factor and oncostatin [58]. Since STAT3 is a transcription factor known to upregulate many of these cytokines (such as IL-6 and IL-10), a vicious cycle of sustained STAT3 activation and excessive production of STAT3-stimulating cytokines often exists in tumors [2,58]. Previous studies have shown that stromal cells present in the tumor microenvironment are also participants of this vicious cycle. Multiple myeloma serves as an example in this regard. Specifically, IL-6—mediated STAT3 activation has been shown to promote the survival of myeloma cells via up-regulating the expression of several survival genes [59]; intriguingly, activation of STAT3 is observed in bone marrow stromal cells present in multiple myeloma, which produce IL-6 to sustain STAT3 activation in myeloma cells [60]. 

Constitutive activation of STAT3 in cancer cells also can be attributed to the expression of various oncogenic protein tyrosine kinases (PTKs). The oncogenic properties of these PTKs stem from the fact that they have escaped the normal cellular control due to a variety of reasons, such as gain-of-function mutations, gene amplifications or chromosomal translocations. One well-known oncogenic PTK is Src, which is known to be over-active in cancer cells. Normally, the activation status of Src is increased by de-phosphorylation of Y527 and phosphorylation of Y416. In cancer cells, de-phosphorylation of Y527 can be due to the activity of tyrosine phosphatases (such as PTP1B), Y527F mutation or deletion of Y527 [61,62]. Phosphorylation of Y416, which correlates with Src activation and its malignant transforming ability, can be found in cancer cells [61,62]. Src has been shown to activate STAT3, and multiple studies have shown that the gene network regulated by STAT3-mediated transcription is required for v-src-induced cellular transformation [14,16,63].

STAT3 is also known to be highly activated in ALK-positive anaplastic large cell lymphoma by the oncogenic fusion protein, NPM-ALK, a constitutively active tyrosine kinase resulted from the specific chromosomal translocation that fuses the *anaplastic large cell lymphoma kinase* (*ALK*) gene on *2p23* to the *nucleophosmin* (*NPM*) gene on chromosome *5q35* [64,65]*.* In this type of lymphoma, NPM-ALK binds to, phosphorylates and activates STAT3, which has been shown to be central to the NPM-ALK—mediated tumorigenesis [66,67,68,69]. In one study, immortalized mouse embryonic fibroblasts with intact STAT3 expression were transformed by NPM-ALK, whereas *STAT3* gene knockout dramatically decreased the malignant transformation by NPM-ALK [66].

Gain-of-function mutations involving JAKs have been implicated in activating STAT3 in specific types of cancer. JAK2-V617F and other JAK mutants are known to activate STAT3 and contribute to the pathogenesis of chronic myeloproliferative neoplasms and leukemias [70,71,72,73]. Mutations in the kinase domain of the epidermal growth factor receptor also have been reported to sustain STAT3 activation by promoting IL-6 production in lung cancer cells [74]. In glioblastoma, a constitutively active mutant of epidermal growth factor receptor was found, and this mutant contributes to and sustains STAT3 activation by inducing a cytokine circuit involving IL-6 and leukemia inhibitory factor, which in turn activates gp130 in the neighboring cells that harbor wild-type epidermal growth factor receptor, leading to an enhanced growth of the entire tumor [75].

#### 2.2.3. Positive Feedback Loops that Sustain Persistent STAT3 Activation

As mentioned above, constitutive STAT3 activation and loss of SHP-1 in ALK-positive anaplastic large cell lymphoma have provided an example of a positive feedback loop. In this section, we will summarize the findings of a number of more recent studies focusing on the autocrine and/or paracrine IL-6/STAT3 stimulatory pathway that forms a positive feedback loop [76,77,78,79]. Using an inducible model of cellular transformation in mammary epithelial cells, Iliopoulos *et al.* has revealed a novel mechanism by which a transient inflammatory signal can initiate cellular transformation [76]. Specifically, using MCF-10A transfected with an inducible expression vector of *v-src*, the authors found that transient activation of v-src is sufficient to induce transformation in these cells. In this system, STAT3 activated by v-src enhances the transcription of miR-21 and miR-181b-1, both of which lead to activation of NF-κB by targeting and inhibiting the expression of two tumor suppressors PTEN and CYLD. Through multiple pathways, activated NF-κB increases the production of IL-6, which in turn sustains the activation of STAT3 [76]. Interestingly, transient transfection of either of the two microRNA species in this circuit was sufficient to induce a stable transformed state, highlighting the importance of these two microRNA species in this transformation process [76].

Lee *et al.* revealed another positive feedback loop that confers STAT3 with a persistent activation property in cancer cells as well as the immune cells present in the tumor microenvironment [77]. In this scenario, STAT3 transcriptionally promotes the expression of a G protein-coupled receptor for the lysophospholipid sphingosine-1-phosphate, sphingosine-1-phosphate receptor-1, which in turn activates STAT3 by increasing IL-6 production and JAK2 tyrosine kinase activity [77]. This positive feedback loop was found in both tumor cells and tumor-associated stromal cells, and the IL-6 produced by these two types of cells mediates the crosstalk between them, and enables a persistent activation of STAT3 in both cell types. Blocking this positive feedback loop in either cell types was shown to decrease tumor growth and metastasis [77,78].

A few recent studies demonstrated other positive feedback loops that connect STAT3, gene regulation, metabolism, survival and proliferation in cancer cells [80]. In one study, pyruvate kinase M2 (PKM2), a protein that is known to be essential for the Warburg effect and proliferation of cancer cells, was found to activate STAT3 via catalyzing its phosphorylation at Y705 [81,82]. In the initiation of a metabolic switch toward aerobic glycolysis similar to the Warburg effect, constitutively active STAT3 was found to promote HIF-1α transcription [83,84,85], which then directly increases the gene expression of PKM2 expression [86]. The PKM2-STAT3-HIF-1α positive feedback loop was shown to contribute to multiple malignant features of cancer. Similarly, STAT3 was shown to form a reciprocal regulatory loop with Polo-like kinase 1 (PLK1) to enhance the proliferation and survival of esophageal cancer cells [87]. In the context of *Helicobacter pylori*-associated gastric cancer, the existence of a positive feedback loop between STAT3 and COX-2 was also demonstrated [88].

#### 2.2.4. Constitutively Active Somatic STAT3 Mutations

Recently, somatic mutations in STAT3 were discovered in hepatocellular adenomas and many types of hematopoietic malignancies, such as T-cell large granular lymphocytic leukemia (T-cell LGL), chronic lymphoproliferative disorders of natural killer cells (CLPD-NKs), diffuse large B-cell lymphoma, and CD30+ T-cell lymphomas [89,90,91,92,93,94,95]. Specifically, Pilati *et al.* identified seven STAT3 mutations in 6/114 hepatocellular adenomas examined. Notably, all six of these tumors were inflammatory hepatocellular adenomas [89], suggesting the specificity of somatic STAT3 mutations for this type of hepatocellular tumor. Somatic STAT3 mutations have also been shown to be frequent in T-cell LGL and CLPD-NKs. Four independent studies have revealed somatic STAT3 mutations in T-cell LGL [90,91,92,93], and the reported percentages of cases carrying STAT3 mutations were 4/36 (11%), 33/120 (28%), 31/77 (40%) and 40/55 (73%), respectively. Two independent studies in CLPD-NKs have described the finding of somatic STAT3 mutations occurring in 3/7 (43%) and 15/50 (30%) of the cases, respectively [91,93]. It is notable that mutations in Y640 and D661 were shown to account for the vast majority of somatic mutations in the *STAT3* gene in both T-cell LGL and CLPD-NKs, representing about 80% of all mutations detected [90,91,93]. 

Intriguingly, most of the STAT3 mutations discovered (e.g., Y640F, D661H, D661V, D661Y, and N647I) reside in the SH2 domain that normally directs STAT3 dimerization, and many of these mutations were suggested to induce amino acid changes that confer higher hydrophobicity to the STAT3 SH2 dimerization surface, potentially facilitating phosphorylation of STAT3^Y705^ and thus the activation of STAT3 [89,90]. Correlating with this concept, both the STAT3-Y640F and STAT3-D661V mutants were shown to increase the transcriptional activity of STAT3 in T-cell LGL, leading to the up-regulation of the downstream target genes of the STAT3 pathway including *IFNGR2*, *BCL2L1* and *JAK2* [90]. Moreover, Y640F, one of the most common STAT3 mutations, was shown to allow homodimerization of STAT3 independent of IL-6 or enhance the STAT3 homodimerization in response to IL-6 [89,90]. Recently, a M206K mutation that localizes in the coiled-coil domain of STAT3 was discovered in diffuse large B cell lymphoma, and this mutation was demonstrated to enhance both the STAT3^Y705^ phosphorylation and its transcriptional activity [94]. Compared with cells harboring wild-type STAT3, STAT3-M206K mutant cells were resistant to the JAK2 inhibitor TG101348, suggesting that this STAT3 mutant possesses constitutive activity [94]. 

### 2.3. The Canonical and Non-Canonical STAT3 Pathways in Cancer

#### 2.3.1. Canonical Mechanisms

**Figure 1:**
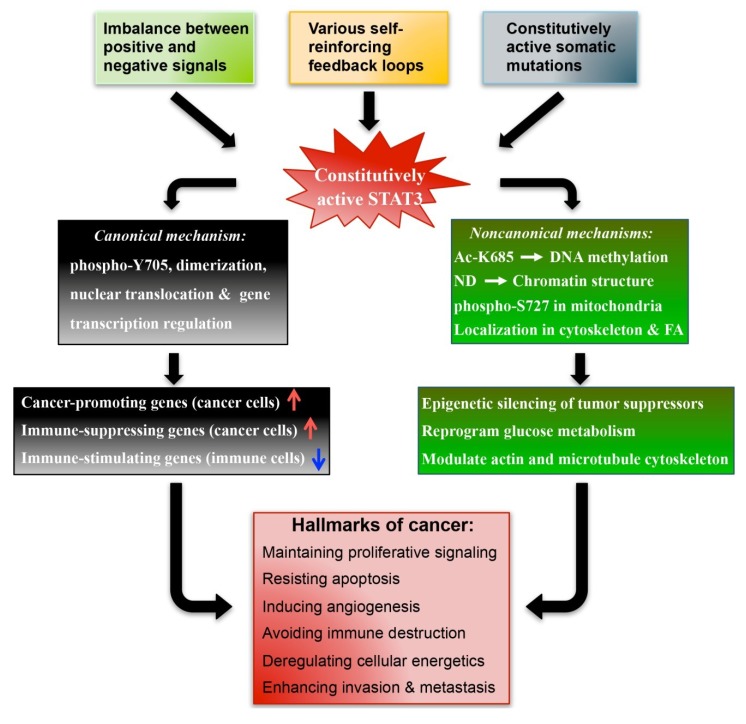
The upstream and downstream mechanisms underlying the pathobiological function of constitutively active STAT3 in cancer. Abbreviations: Ac, acetylation; ND: N-terminal domain; FA, focal adhesion.

In the canonical pathway, the oncogenic function of STAT3 is dependent on its phosphorylation at Y705, and the subsequent dimerization and nuclear translocation [96]. As a transcription factor, STAT3 directly regulates the expression of a wide spectrum of genes, many of which play key roles in various aspects of oncogenesis (Figure 1).

Cell proliferation: It is well known that STAT3 increases the transcription and expression of multiple gene targets that are crucial in the regulation of cell cycle progression. These gene targets include *Cyclin D1*, *c-Myc*, *PLK-1* and *Pim1/2* [4,87]. Thus, inhibition of STAT3 signaling using siRNAs or pharmacological agents can effectively reduce tumor growth by suppressing the expression of these cell-cycle facilitators [26,97]. A specific example is the observation that enforced expression of the STAT3-DN construct suppressed the proliferation of head and neck squamous cell carcinoma cells by lowering the expression of Cyclin D1 [98]. Zhang *et al.* also showed that siRNA-mediated STAT3 knockdown in esophageal squamous cell carcinoma cells repressed cell proliferation and tumor growth in mice by suppressing PLK1 expression [87].

Resistance to apoptosis: STAT3C has been shown to promote the survival of tumors cells in various models [1,4]. To achieve this, STAT3 regulates both the intrinsic and extrinsic apoptotic pathways. In many cancer cell types, STAT3 can transcriptionally increase the expression of various anti-apoptotic proteins involved in the intrinsic apoptotic pathway, such as survivin and the Bcl-2 family members (e.g., Bcl-xL, Bcl-2 and Mcl-1) [1,4]. STAT3 has been shown to cooperate with c-Jun to suppress the expression of FAS, a crucial mediator of the extrinsic apoptotic pathway; multiple studies have demonstrated that activated STAT3 protects cancer cells from FAS ligand—induced apoptosis and p53-dependent apoptosis [99,100,101,102].

Induction of angiogenesis: STAT3 has been shown to augment tumor angiogenesis in multiple cancer types, such as melanoma, pancreatic cancer, cervical cancer and renal carcinoma [103,104,105,106,107,108]. Mechanistic studies have revealed that STAT3 directly binds to the promoter region of the *VEGF* gene and promotes its transcription, thereby enhancing tumor growth and metastasis [103,104,105]. Consistent with these findings, a significant correlation between evidence of STAT3 activation and VEGF expression was observed in cell lines derived from breast cancer, head and neck carcinoma, melanoma and pancreatic cancer [103,104,105]. Furthermore, ectopic expression of STAT3C in B16 melanoma cells was found to promote the formation of capillaries in the xenografts established in nude mice [105]. The STAT3-VEGF circuit involves more than cancer cells within tumors. Using the inducible STAT3 knockout mouse model, a recent study has shown that STAT3 promotes the production of angiogenic factors (including VEGF and bFGF) in myeloid-derived suppressor cells and macrophages present in the tumor microenvironment, thereby stimulating endothelial cell migration and tumor angiogenesis [107]. Apart from being a stimulator of VEGF production, STAT3 also has been shown to directly mediate the pro-angiogenic activity of VEGF in microvascular endothelial cells [107,109,110]. For example, Yahata *et al.* found that VEGF stimulates STAT3 phosphorylation and nuclear translocation in human dermal microvascular endothelial cells, and inhibition of STAT3 using a dominant negative construct significantly impaired VEGF-induced migration and tube formation of these cells [109]. 

Promotion of invasion and metastasis: Previous studies have shown that STAT3 can promote invasiveness and the metastatic potential of cancer cells. In one study, transfection of *STAT3-DN* in pancreatic cancer cells suppressed tumor growth and liver metastasis in nude mice, whereas transfection of *STAT3C* enhanced tumor growth and liver metastasis [104]. STAT3C may exert these biological effects via several different mechanisms. First, STAT3 can trigger epithelial to mesenchymal transition (EMT) by upregulating several key EMT regulators such as Twist-1, Snail and ZEB-1 [111,112,113,114]. Second, STAT3 is known to increase the expression of various matrix metalloproteinases (MMPs), including MMP-1, MMP-2, MMP-7 and MMP-9, which facilitate cancer cell invasiveness by degrading various extracellular matrix proteins [15,115,116,117,118,119,120]. Third, STAT3 can directly enhance the expression of focal adhesion molecules, such as integrin α6 and CTEN (C-terminal tensin-like) [16,121]. These observations are biologically significant, since over-expression of focal adhesion related proteins, which connect the cellular cytoskeleton to the extracellular matrix, has been linked to cancer metastasis [122,123]. Lastly, in a recent report, cytokines (e.g., IL-6 and IL-10) produced by tumors cells in a STAT3-dependent manner were shown to activate STAT3 in myeloid cells in the tumor microenvironment, resulting in sustained activation of STAT3 in these myeloid cells; these myeloid cells circulate to the lungs and promote the formation of pre-metastatic niche to support future cancer metastasis [78]. 

Evasion of anti-tumor immunity: The concept that STAT3 has a role in dampening the anti-tumor immune response came from the observation that tumor cell death induced by STAT3 blockade is associated with infiltration of various immune effector cells [124]. Several subsequent studies also have implicated STAT3 in the context of tumor immuno-surveillance [1]. For example, blocking STAT3 in macrophages has been shown to activate anti-tumor immune responses in a murine model of breast cancer [125]. Mechanistically, STAT3 signaling was shown to inhibit TH1-type inflammation after lipopolysaccharide stimulation by suppressing the production of specific cytokines and nitric oxide [126]. Furthermore, the STAT3 activity in tumor cells enhances the expression of several immune-suppressing soluble factors, such as IL-6, IL-10 and VEGF, all of which are known to prevent the maturation of dendritic cells [1,125]. STAT3 activation in immature dendritic cells has been shown to impair the expression of MHC class II molecules, CD80, CD86 and IL-12, thereby hindering their maturation and thus decreasing their ability to promote the anti-tumor function of CD8-positive T cells and natural killer cells [1].

STAT3 and cancer stem cells: In recent years, accumulating evidence suggests that STAT3 carries a critical role in promoting the self-renewal of cancer stem cells [18,19,20,21,22]. In one study, transfection of *STAT3C* in glioblastoma cells was found to increase the expression of several stem cell factors, such as Sox2, Oct4 and Nanog [19]. Using a large-scale loss-of-function screen, Marotta *et al.* identified 15 genes that are required for the proliferation of the breast cancer stem cell population characterized by the CD44^+^CD24^−^ immunophenotype, and five of these genes facilitate STAT3 activation [20]. Further investigation indicated that the IL-6/JAK2/STAT3 pathway was preferentially active in CD44^+^CD24^−^ breast cancer stem cells, and inhibition of JAK2 decreased the number of cancer stem cell number and blocked the growth of xenografts in mice [20]. In glioblastoma, it has been shown that the stem cell factor EZH2 interacts with STAT3 and tri-methylates its K180 residue, thereby activates STAT3 and promotes tumorigenesis [21]. Importantly, the EZH2-STAT3 interaction preferentially occurs in the stem cell population, suggesting a specific role of these two factors in maintaining cancer stemness [21]. In another study, it was found that STAT3 activation by BMX (bone marrow X-linked), a non-receptor tyrosine kinase, is required for maintaining the self-renewal and tumorigenic potential of cancer stem cells in glioblastoma [19]. 

#### 2.3.2. Non-Canonical Mechanisms

The non-canonical pathway comprises a number of biological functions of STAT3 that have been shown to be independent of its transcription activity or the phosphorylation of STAT3^Y705^. 

*Gene silencing and regulation*: Zhang *et al.* discovered that STAT3 interacts with DNA methyltransferase 1 (DNMT1) and histone deacetylase 1 (HDAC1), by which STAT3 facilitates gene methylation and silencing of *SHP-1* in malignant T lymphocytes; furthermore, blocking the expression of either DNMT1 or STAT3 using siRNA was found to induce DNA demethylation and re-expression of *SHP-1* in these cells [53]. In another study, it was revealed that K685-acetylated STAT3 cooperates with DNMT1 to silence several tumor suppressor genes, including *TP53*, *SHP-1*, *SOCS3* and *CDKN2A*, in melanomas; mutation of STAT3^K685^ or treatment with resveratrol (a histone deacetylase activator) was found to diminish the tumor-promoting function of STAT3 in melanoma [127]. Yuan *et al.* demonstrated that histone acetyltransferase p300 is responsible for STAT3 acetylation at K685, and this process can be reversed by histone deacetylase 1 [128]. Recently, it also was shown that nuclear localized CD44 facilitates STAT3 acetylation on K685 residue [129].

Previous studies have provided multiple lines of evidence that STAT3 can exert oncogenic functions that are independent of the phosphorylation of its Y705 residue. First, un-phosphorylated STAT3 has been found to migrate to the nucleus with the help of importin-α3 [130]. Second, it has been shown that both un-phosphorylated STAT3 and STAT3-DN can interact with NFκB in the nucleus to drive the expression of multiple cancer-related genes, such as *RANTES*, *IL-6*, *IL-8*, *MET* and *MRAS* [131,132]*.* Third, in prostate cancer and chronic lymphocytic leukemia, phosphorylation of STAT3^Ser727^ rather than STAT3^Y705^ was found to be crucial for the nuclear translocation, DNA binding and the tumor-promoting function of STAT3. This finding correlates well with the observation that STAT3-DN does not interfere with the oncogenic function of STAT3 in these experimental models [133,134]. Recently, Timofeeva *et al.* showed that un-phosphorylated STAT3 binds to the regulatory region of several pro-apoptotic genes (such as *FOS*, *CHOP* and *NR4A2*) in tumor cells and prevents their expression by promoting a repressive chromatin structure [135]. Correlating with this concept, the authors were able to show that the observed oncogenic effect of STAT3 was independent of its phosphorylation at Y705, whereas the N-terminal domain of STAT3 is indispensable, because the N-terminal domain was shown to be required for the dimerization of un-phosphorylated STAT3. In another study by the same group of researchers, it was found that un-phosphorylated STAT3 can bind to the interferon γ-activated sequence (GAS) either as dimers or as monomers, and it regulates gene expression via its binding to AT-rich DNA sequences and regulation of chromatin structure [136]. 

*Mitochondrial STAT3*: In 2009, two *Science* papers simultaneously reported the function of STAT3 present in the mitochondria [137,138]. Specifically, it was demonstrated that mitochondrial STAT3 controls cell respiration and metabolism by enhancing the activity of succinate oxidoreductase (complex II), ATP synthase (complex V) and lactate dehydrogenase, thereby sustaining the glycolytic and oxidative phosphorylation activities that are characteristic of cancer cells [138]. In one of these two papers, it was demonstrated that the phosphorylation of STAT3^S727^ rather than STAT3^Y705^ is required for the oncogenic role of mitochondrial STAT3 in the context of H-ras—induced transformation [137]. More recently, the significance of mitochondrial STAT3 also has been documented in breast cancer, in which it promotes tumor growth and metastasis by suppressing the generation of reactive oxygen species; again, this biological effect is dependent on phosphorylation of STAT3^S727^ but not that of STAT3^Y705^ [139]. 

*STAT3 modulates cytoskeleton and focal adhesions*: Results from multiple studies support the concept that STAT3 can modulate the cytoskeletal structures of the cells via multiple mechanisms. As mentioned above, STAT3 has been shown to regulate the migration and invasiveness of cancer cells by transcriptionally up-regulating the expression of focal adhesion-associated proteins [16,121]. Intriguingly, STAT3 also has been shown to directly localize in focal adhesion sites in ovarian cancer cells by interacting with multiple focal adhesion-associated molecules, such as focal adhesion kinase (FAK) and Paxillin [140,141]. In other studies, STAT3 was found to play an important role in regulating the assembly of cytoskeleton network, including actin and microtubule cytoskeleton, thereby promoting cell migration and invasion [142,143,144]. Specifically, gene transfer of wild-type *STAT3* was found to promote the rearrangement of actin stress fibers and microtubules, and facilitates the formation of lamellipodia in prostate cancer cells [142,143]; in the same studies, it was demonstrated that enforced expression of STAT3 can increase cell migration *in vitro*, and substantially enhance lung metastasis *in vivo* [142,143]. In other studies, STAT3 was shown to promote microtubule polymerization by interacting with and antagonizing the function of Stathmin, a tubulin-associated protein that modulates the polymerization of microtubules [145,146]. A recent study also has shown that depletion of STAT3 in gastric cancer cells impairs microtubule polymerization due to a disruption of the interaction between STAT3 and Stathmin; as a result, cell migration and invasion were decreased [144]. 

## 3. The Tumor Suppressor Functions of STAT3

### 3.1. STAT3 Can Exert Tumor Suppressor Effects

**Figure 2:**
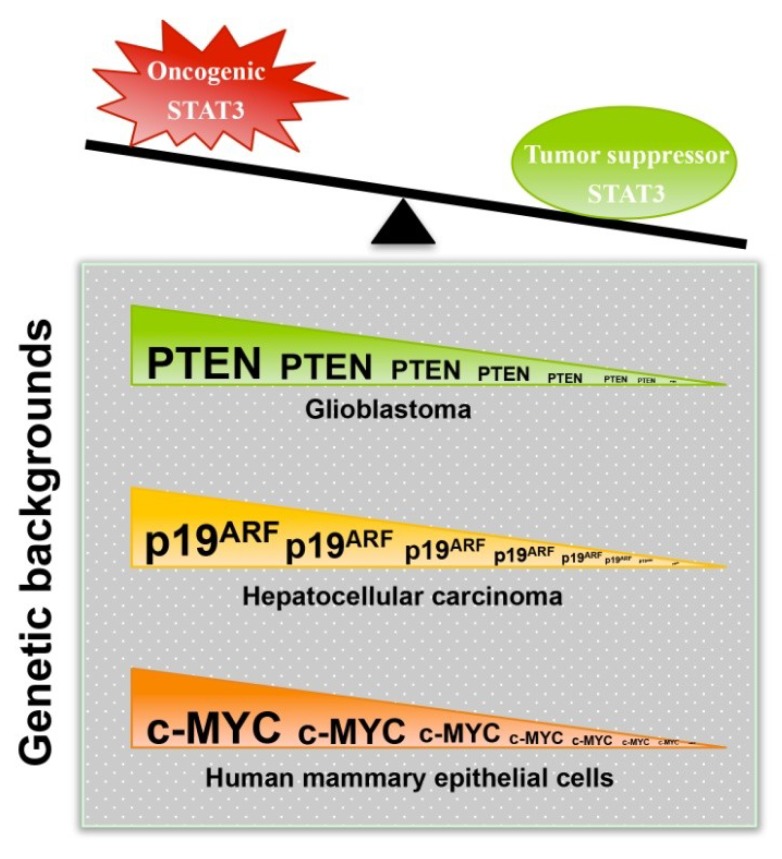
The genetic background determines whether STAT3 is oncogenic or tumor suppressive.

While the oncogenic effects of STAT3 have been well recognized, a relatively small number of studies published previously have shown that STAT3 carries tumor suppressor functions, a seemingly paradoxical notion. Importantly, some of these studies have proposed a novel concept that STAT3 can function as an oncoprotein or tumor suppressor in the same cells, and the decision is dependent on the genetic background and/or coexisting biochemical defects (Figure 2). This section summarizes these experimental findings.

The first experimental evidence to support that STAT3 carries tumor suppressor functions comes from a study published in 2008 [147]. Using astrocytes derived from conditional STAT3 knockout mice, the authors found that the simultaneous deletion of STAT3 and shRNA knockdown of PTEN resulted in a dramatic increase in cell proliferation *in vitro* and tumor formation in SCID mice, whereas siRNA knockdown of PTEN alone (*i.e.*, in the presence of normal STAT3 expression) resulted in significantly less tumorigenic effects in these cells. Furthermore, no significant tumor suppressor effect of STAT3 was observed in the presence of normal PTEN expression. In other words, in this experimental model, the tumor suppressor effects of STAT3 were revealed only in the absence of PTEN expression. Interestingly, in the same study using astrocytes harvested from the same conditional STAT3 knockout mice, the authors also found that transfection of EGFRvIII (epidermal growth factor receptor type III variant) in STAT3^+/+^ astrocytes resulted in tumor formation in SCID mice, whereas the same treatment did not result in any tumor formation in STAT3^−/−^ astrocytes. Thus, the oncogenic effect of STAT3 was found to be dependent on the co-existence of EGFRvIII, which was shown to complex with STAT3 in the nuclei of these cells. Taken together, this study has demonstrated that STAT3 can function as a tumor suppressor as well as an oncoprotein, and the genetic background and/or coexisting biochemical defects of the cells play a key role in determining the functions of STAT3. 

Another report describing the tumor suppressor effects of STAT3 was published in 2011 [148]. Using ras-transformed mouse hepatocytes harvested from homozygous *p19^ARF^* knockout mice, the authors found that transfection of *STAT3* or *STAT3C* significantly suppressed tumorigenecity in a SCID mouse xenograft model. In comparison, transfection of a double *STAT3* mutant in which both Y705 and S727 cannot be phosphorylated led to significant tumor growth in SCID mice. In the same paper, the authors also found that cells transfected with the double *STAT3* mutant resulted in liver and lung metastasis after intravenous injection of the cells, while transfection with *STAT3* or *STAT3C* significantly decreased the metastatic potential of these hepatocytes. While the mechanisms underlying these observations require further investigations, these results have suggested the relevance of ras and/or p19^ARF^ in regulating the tumor suppressor function of STAT3. 

In another study, the tumor suppressor function of STAT3 was revealed in the Apc(Min/+) mouse model of colorectal cancer [149]. In this model, the oncogenic driving force was provided by the *multiple intestinal neoplasia* (*Min*) gene, which is essentially the murine *Apc* (*adenomatous polyposis coli*) gene carrying a nonsense mutation at codon 850, leading to the production of a truncated and non-functional Apc protein. By crossing these animals with conditional STAT3 knockout mice, the authors were able to generate mice with which they assessed the impact of STAT3 knockout in the Apc(Min/+)-carrying intestinal epithelial cells. While deletion of STAT3 in the intestinal epithelial cells reduced the multiplicity of early adenoma formation (*i.e.*, oncogenic role), ablation of STAT3 in the later stage of tumor progression significantly increased the invasiveness of the tumors and decreased the survival of the animals (*i.e.*, tumor suppressor role). 

The last example came from a recent study examining drug-induced liver carcinogenesis in conditional STAT3 knockout mice. It was found that the hepatocytes with STAT3 expression had a significantly less tumor formation induced by chronic carbon tetrachloride, as compared to hepatocytes with STAT3 knockout. In contrast, hepatocytes with STAT3 expression were found to have a significantly higher tumor formation induced by diethylnitrosamine, as compared to hepatocytes with no STAT3 expression [150]. In other words, STAT3 can be either oncogenic or tumor suppressive, depending on the use of different carcinogens. As stated by the authors, the two carcinogens used in this study likely induce liver carcinogenesis through different mechanisms. Specifically, chronic carbon tetrachloride is believed to induce liver cancer by causing chronic liver injury, inflammation and fibrosis, whereas diethylnitrosamine is believed to induce liver cancer by promoting the formation of alkylated DNA adducts after being metabolically activated by cytochrome P450 enzymes in the liver [151]. As the author speculated, the tumor suppressor function of STAT3 in the carbon tetrachloride model correlates well with the fact STAT3 is known to play an important role in protecting liver against hepatocellular damage. In contrast, in the diethylnitrosamine model, the authors believed that the oncogenic effects of STAT3 are due to the fact that STAT3 can increase the expression of cyclin D1 and suppress the expression of p21. Thus, STAT3 can be a friend or foe, depending on the pathogenesis of specific types of cancer. 

### 3.2. Mechanisms That Mediate or Regulate the Tumor Suppressor Function of STAT3

Several recent studies have shed insights into how STAT3 might mediate tumor suppressor effects. In one study using thyroid cancer cell lines and an SCID mouse xenograft model, Couto *et al.* found that siRNA knockdown of STAT3 resulted in significantly increased tumor growth, and this observation correlated with increased glucose consumption, lactate production, and expression of HIF-1α target genes in the tumor cells [152]. These findings suggest that one of the mechanisms by which STAT3 inhibits tumorigenesis is mediated by inhibiting aerobic glycolysis in the tumor cells. 

Several proteins have also been implicated in regulating the function of STAT3 in cancer cells. In one study, STAT3C was found to suppress cell growth in p53^−/−^ mouse embryonic fibroblasts transformed by c-Myc, but exert no appreciable effects on the same type of fibroblasts that were transformed by ras [153]. In another study, c-Myc was shown to function as a molecular switch to alter the function of oncostatin M-activated STAT3 in human mammary epithelial cells deficient in both p53 and p16. Specifically, oncostatin M-STAT3 signaling was found to suppress c-Myc expression and tumorigenesis in human mammary epithelial cells that were deficient in both p53 and p16 [154]. In contrast, when c-Myc expression was restored by transfecting a constitutively active c-Myc construct into the cells, the cellular response to the oncostatin M-STAT3 signaling was switched from tumor suppressive to tumor promoting. Thus, in this particular cell context (*i.e.*, deficiency in both p53 and p16), it appears that c-Myc is the dominant oncogenic protein, and STAT3 is a tumor suppressor. 

In mice generated by crossing the Apc(Min/+) mice and conditional STAT3 knockout mice, Lee *et al.* found that that ablation of STAT3 significantly increase the invasiveness of colorectal cancer [155], a finding that is in parallel to that reported by Musteanu [149]. Furthermore, the authors found that STAT3 mediates the tumor suppressor effects by binding to GSK3β, which in turn promotes the phosphorylation and the degradation of Snail, a critical regulator of the EMT and cancer metastasis.

### 3.3. Clinical Observations Supporting the Tumor Suppressor Role of STAT3

While the majority of studies evaluating the prognostic value of STAT3 and/or pSTAT3 have pointed to its oncogenic effects, a few studies have reported contradictory observations, with the expression of STAT3/pSTAT3 found to be “paradoxically” associated with a better prognosis in various types of cancer, including those of the head and neck, salivary gland, breast, nasopharynx and rectum. For instance, high expression of pSTAT3 or nuclear STAT3 in head and neck cancer was found to be associated with a favorable clinical outcome; the progression free survival for patients carrying tumors with high expression of nuclear STAT3 was significantly longer than that of patients carrying tumors with relatively low STAT3 expression [156]. In another study including a large cohort of patients with salivary gland tumors, patients carrying tumors with strong nuclear pSTAT3 immunostaining were found to have a better clinical outcome compared with those carrying tumors with moderate or weak nuclear pSTAT3 staining; moreover, strong nuclear pSTAT3 also significantly correlated with a low histologic grade, as well as the absence of lymph node and distant metastases [157]. In breast cancer, two studies have documented that nuclear pSTAT3 expression significantly correlated with a favorable clinical outcome, although this correlation was restricted to patients with low-grade tumors or node-negative tumors [158,159].

It is important to point out that the contradiction regarding the prognostic significance of STAT3 does not appear to be due to cell-type specificity. To illustrate this point, STAT3 was reported to be a marker of worse clinical outcome in head and neck cancer as well as breast cancer [15,160,161,162,163], the same types of cancer in which STAT3 was found to be associated with a better clinical outcome in other studies [156,157,159]. While these discrepancies may be partly attributed to the use of slightly different immunohistochemical methods and/or the inclusion of different patient cohorts, one may consider an alternative possibility, in light of the recent experimental data showing that the genetic background and/or coexisting biochemical defects can dictate whether STAT3 exert oncogenic or tumor suppressor effects in cancer cells. As mentioned in Section 3.1, the expression and/or functional status of PTEN, p53, p19^ARF^ and c-myc have been implicated in influencing the function of STAT3 in various experimental models. With this new knowledge, it is perceivable that the studies of different cohorts of tumors that are biased toward specific molecular profiles will lead to different conclusions regarding the prognostic significance of STAT3. 

## 4. STAT3β and the Tumor Suppressor Effects of STAT3

### 4.1. STAT3β Has Biochemical and Biological Features Different from STAT3α

**Figure 3:**
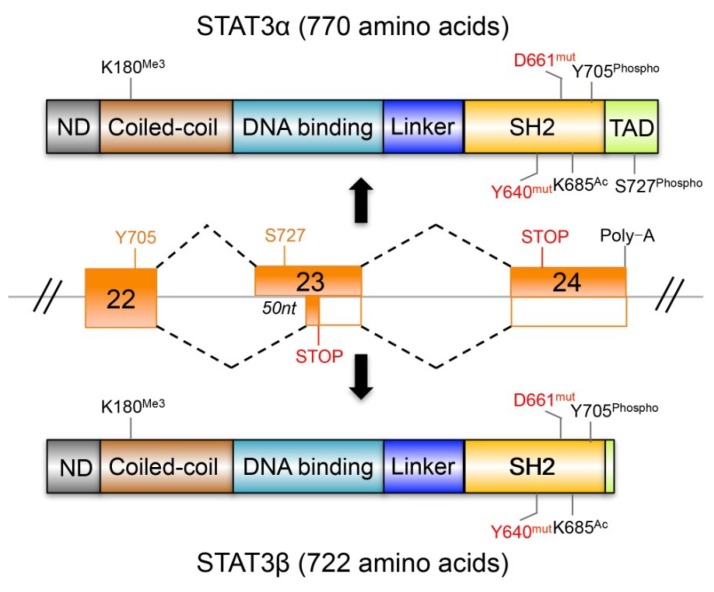
A schematic diagram showing the generation of STAT3α and STAT3β from primary STAT3 mRNA by alternative splicing, and the modification sites and somatic mutation sites in STAT3 that are relevant to cancer. Abbreviations: ND, N-terminal domain; SH2, Src homology 2; TAD, transactivation domain; Me3, trimethylation; mut, mutation; Phospho, phosphorylation; Ac, acetylation.

The *STAT3* transcript generates two isoforms, STAT3α and STAT3β, which are generated by alternative splicing of exon 23. The relationship of these two isoforms and the initial characterization of STAT3β have been previously described [164]. Briefly, STAT3β is a result of the deletion of the first 50 nucleotides of exon 23, leading to a frame shift that introduces 7 amino acid residues followed by a stop codon. Thus, STAT3β is a truncated version of STAT3α, with the two isoforms sharing the identical amino acid sequence except for the 55 amino acids at the C-terminal of STAT3α that are replaced with a unique 7-amino-acid sequence in STAT3β. Consequently, STAT3β lacks the transactivation domain (Figure 3). 

The expression STAT3β is found ubiquitously, but its level is lower than that of STAT3α [164,165]. Intriguingly, the ratio of the expression levels of STAT3α and STAT3β changes in response to physiologic changes or specific cytokine stimulations. For instance, the expression of STAT3β has been shown to dramatically increase in mouse hepatocytes in response to lipopolysaccharide-induced endotoxin shock [166]. The expression of STAT3β was also found to be increased in myeloid cells as they differentiate [167,168,169].

Previous studies have shown that STAT3α and STAT3β have significantly different biochemical and biological properties. Some of the early studies have showed that phosphorylated STAT3β^Y705^ (or pSTAT3β) has a much longer half-life and nuclear retention than phosphorylated STAT3α^Y705^ (or pSTAT3α) [170,171,172]. The difference in their biological behaviors was also highlighted in a study, in which STAT3β was found to have a higher DNA binding ability than STAT3α in COS-7 cells that had been serum starved [172]. Correlating with these observations, a subsequent study revealed that the negatively charged 55 amino acids present in the C-terminus of STAT3α confer a decreased stability of the STAT3α dimers, and this difference in the dimer stability between STAT3α and STAT3β is believed to be responsible for the longer half-life and nuclear retention of pSTAT3β [173]. Another study showed that the unique C-terminal 7-amino acid domain of STAT3β also contributes to the increased nuclear retention of STAT3β, since with the deletion of this domain was shown to decrease the nuclear retention time of STAT3β [170]. Moreover, compared with STAT3α, STAT3β showed an approximate of two-fold decrease in intra-nuclear mobility, and this difference is more obvious under IL-6 stimulation [170]. 

### 4.2. The Dominant Negative Role of STAT3β

Without the STAT3 transactivation domain, STAT3β is expected to be unable to activate promoters carrying the interferon/IL-6—responsive element. With this in mind, it was postulated that STAT3β might function as a dominant negative factor that can somewhat neutralize and/or regulate the biological effects of STAT3α. Experimental data supports this concept, as STAT3β was found to inhibit the transcription activity of full-length STAT3 or STAT3α [164]. In other studies, it has been demonstrated that STAT3β can abolish the transcriptional activation of several STAT3 downstream targets including *Cyclin D1*, *Bcl-xL* and *Mcl-1*, leading to inhibition of tumor growth and promotion of apoptosis [174,175,176]. While STAT3α was also found to act in cooperation with c-Jun to suppress the transcription of *Fas*, it was shown that STAT3β could counteract the transcription suppressive function of STAT3α in this context [99]. In the literature, we are able to find several studies in which STAT3β was used as an experimental tool to block STAT3 signaling [124,177,178].

### 4.3. STAT3β Regulates a Gene Set That Is Distinct from That of STAT3α

In addition to its dominant negative role, STAT3β is believed to carry other functions, considering the fact that it possesses most of the important STAT3 functional domains including the activation domain (*i.e.*, which carries the tyrosine 705 residue), the Src homology2 (SH2) domain that is responsible for STAT3 dimerization and its binding to the receptor complex (e.g., JAKs), the coiled-coil domain that allows STAT3 to interact with other proteins, and the DNA binding domain. Accumulating evidence is in support of this concept. Schaefer *et al.* reported that STAT3β can cooperate with c-Jun to activate a promoter containing the IL-6 responsive element; interestingly, the nuclear extract from cells transfected with the *STAT3β* cDNA, but not that from cells transfected with the *STAT3α* cDNA, formed a complex with an oligonucleotide containing the STAT3 binding site [165]. In another study, STAT3β was found to compensate the function of STAT3; specifically, transfection of STAT3β effectively induced the expression of acute phase genes in STAT3-null hepatocytes challenged with lipopolysaccharide [179]. Furthermore, it has been demonstrated that STAT3β can rescue the embryonic lethality in STAT3-null mice, and high expression of endogenous STAT3β (compared to STAT3α expression) did not impair the activity of STAT3α in transgenic mice, either in embryos or in adult mice [180]. Another study showed that, compared with the wild-type mice, mice with specific STAT3β ablation were hypersensitive to lipopolysaccharide-induced inflammation, and these findings correlated with the dramatic alterations of the expression of lipopolysaccharide-responsive genes in the hepatocytes [166]. 

More recently, it has become evident that STAT3β has its own set of target genes that is distinct from that of STAT3α, and these genes include *LEDGF* (lens epithelium-derived growth factor), *PCAF* (p300/CBP-associated factor), CCNC (cyclin C), *PEX1* (peroxisomal biogenesis factor 1) and *STAT1β* [181]. In the same study, a novel agent called morpholino was also described; specifically, morpholino was designed to modulate the STAT3 alternative splicing process such that STAT3β is favored at the expense of STAT3α. The shift from STAT3α to STAT3β was found to dramatically decreased tumor growth, which was shown to be mediated by STAT3β-specific downstream targets [181]. In another study, using STAT3-null murine embryonic fibroblasts with inducible expression of STAT3α or STAT3β, Ng *et al.* identified distinct gene sets modulated by STAT3α and STAT3β, respectively. Specifically, the authors identified 506 genes that are regulated by both STAT3α and STAT3β, with 651 STAT3α-specific target genes and 1331 STAT3β-specific target genes [171]. Taken together, there is strong evidence that STAT3β can function as an independent transcriptional regulator, despite the fact that it lacks the transactivation domain. In the absence of the transactivation domain, it is likely STAT3β relies on its interactions with other transcription co-factors, such as c-Jun, to regulate gene expression [165]. 

### 4.4. STAT3β Regulates the Phosphorylation Dynamics of STAT3α

It was recently found that STAT3β could directly regulate STAT3α. In one study, STAT3β was found to upregulate and prolong the phosphorylation of STAT3α^Y705^ upon stimulation with oncostatin M in murine embryonic fibroblasts [171]. Specifically, in cells with only STAT3α expression, phosphorylation of STAT3α^Y705^ stimulated by oncostatin M was transient, reaching a peak at 15 min after the exposure to oncostatin M exposure and decreasing to an undetectable level at 60 min. In contrast, in the presence of STAT3β, phosphorylation of STAT3α^Y705^ was sustained at a high level for 120 min. Correlating with these findings, cells with STAT3β expression had a prolonged nuclear retention of STAT3. In the same study, it was also demonstrated that an intact SH2 domain is required for the interaction between the two STAT3 isoforms and the cross-regulation of STAT3β on STAT3α.

### 4.5. Is STAT3β Responsible for the Tumor Suppressor Function of STAT3?

How are these findings related to the observation that STAT3 can function as a tumor suppressor? While the answer to this question needs further investigation, one may speculate that heterodimerization of pSTAT3α and pSTAT3β may sequester pSTAT3 away from its DNA target genes or interfere with the transcription activity of pSTAT3 due to the absence of the transactivation domain in STAT3β. Thus, in the presence of a high level of pSTAT3β, pSTAT3α becomes less available or effective in mediating gene transcription, despite the fact that its expression and nuclear retention are higher due to the stabilizing effect of pSTAT3β. Results from one of our recent studies support this concept. Using esophageal squamous cell carcinoma cell lines, we were able to demonstrate that the transfection of *STAT3β* significantly reduced the transcriptional activity of STAT3α, although the levels of pSTAT3α in the cytoplasm and nuclei were increased (data not shown).

Correlating with these findings, STAT3β has been shown to play a tumor suppressor role in various types of cancer, including melanoma, breast cancer and lung cancer [99,124,177,178,181,182]. For instance, transient *STAT3β* transfection in murine B16 melanoma cells induced cell cycle arrest as well as apoptosis [177]. Furthermore, electro-injection of *STAT3β* cDNA into established B16 melanoma xenografts in SCID mice induced apoptosis and suppressed tumor growth [124]. In another study, gene transfer of *STAT3β* was found to result in marked shrinkage of xenografts in SCID mice, whereas siRNA knockdown of STAT3 resulted no significant effect on breast tumor growth [181]. Interestingly, in one study, apoptosis induced by STAT3β was found in *STAT3β*-transfected cells as well as the bystander non-transfected cells in the same tissue culture, due to the production of TRAIL (TNF-related apoptosis inducing ligand) induced by *STAT3β* transfection [177]. Moreover, it has been shown that transfection of *STAT3β* cDNA in a human melanoma cell line increases FAS expression and enhances apoptosis induced by FAS-ligand and UV irradiation [99]. A few years later, the same research team reported that STAT3β can effectively suppress the growth of human melanomas xenografted in nude mice by increasing the expression of TRAIL receptor 2, a pro-apoptotic factor expressed on the cell surface of the tumor cells [182].

## 5. Evaluation of STAT3 Expression in Patient Samples

Since the late 90s when the potent oncogenic effects of STAT3α was demonstrated in various experimental models, there have been numerous published clinicopathologic studies showing a significant correlation between a high expression level of STAT3 and/or pSTAT3 (typically shown using immunohistochemistry applied to archival tissues) and a worse clinical outcome and/or adverse pathologic features. Nevertheless, as mentioned in Section 4, a few clinicopathologic studies had pointed to the opposite conclusion. While some of these discrepancies may be theoretically attributed to differences in the immunohistochemical methods and/or interpretation, we believe that this explanation is not sufficient, since these experimental protocols have been extensively published. Another possibility is that the discrepancies among published studies are related to cell-type specificity, such that STAT3 is oncogenic in one type of cancer whereas it is a tumor suppressor in another. We do not favor this argument, since there are a good number of examples in which studies of the same types of cancer came up to different conclusions (Section 4). 

While we summarized all the existing data regarding STAT3β, we came to realize that virtually all published clinicopathologic studies pertaining the prognostic value of STAT3/pSTAT3 did not differentiate the two STAT3 isoforms. Considering the concept that STAT3 can be an oncoprotein or a tumor suppressor (as described in Section 3), and that STAT3β functions as a dominant negative factor for STAT3α (as described in Section 4), it is highly possible that the expression of STAT3 and/pSTAT3 in tumor samples may correlate with the clinical outcome in either direction, depending on the genetic background of the tumor cells and/or the coexisting biochemical defects present in these cells, and whether the tumor suppressor effects of STAT3β overcomes the oncogenic effects of STAT3α. Thus, a high expression level of pSTAT3 detectable by immunohistochemistry does not necessarily correlate with the transcriptional activity of STAT3, which is believed to be an important determinant of the oncogenic effects of STAT3. In other words, accurate evaluation of the biological and clinical significance of STAT3 in human tumor samples will require some understanding of the relevant coexisting biochemical defects (e.g., c-myc, PTEN and p14^ARF^) as well as the simultaneous evaluation of STAT3α and STAT3β. From a technical perspective, the detection of the two STAT3 isoforms in tumor samples is currently feasible only by analyzing fresh tissues using western blots, with which STAT3α and STAT3β can be differentiated from each other based on the difference in their molecular weights (*i.e.*, with STAT3β being the faster-migrating isoform). Immunohistochemistry, which has been used in the vast majority of clinicopathologic studies of STAT3 in the past, is only useful if antibodies specific for STAT3α or STAT3β (and their phosphorylated counterparts) are available. To our knowledge, most if not all commercially available anti-STAT3 or anti-pSTAT3 antibodies are not isoform-specific. We are aware of a small number of reports describing antibodies that recognize STAT3β but not STAT3α, although these antibodies are not widely available and/or extensively characterized. 

## 6. Conclusions

Since the initial description of the oncogenic role of STAT3 in the late 90s, much has been learned about the significance and the divergent roles of STAT3 in cancer biology. The experimental results summarized in this review have highlighted the concept that STAT3 can be friend or foe in cancer, and there are a relatively large number of factors that are known to dictate how STAT3 affects the malignant phenotypes and clinical behaviors of cancer. It is clear that the roles of STAT3 in cancer cells are substantially more complicated that it was appreciated previously. We believe that this new knowledge carries significant implications in how we should evaluate the prognostic value of STAT3 in tumors, and in how we design new anti-cancer drugs targeted against STAT3. More attention should be given to factors or processes that have not been discovered or appreciated until recently, including the non-canonical mechanism of STAT3, acetylation of STAT3, the dominant negative effects of STAT3β, as well as specific genetic background and/or coexisting biochemical defects that might functionally interact with STAT3. It is our hope that researchers in this field will take this new knowledge into consideration in their future studies.
